# Vulvar Crohn disease: A case report

**DOI:** 10.1177/2050313X251350341

**Published:** 2025-07-07

**Authors:** Jason Kreutz, Colton Jensen, Marlene Dytoc

**Affiliations:** 1 Cumming School of Medicine, University of Calgary, AB, Canada; 2 Division of Dermatology, Faculty of Medicine, University of Alberta, Edmonton, AB, Canada

**Keywords:** vulvar Crohn’s disease, cutaneous Crohn’s disease, metastatic Crohn’s disease

## Abstract

Vulvar Crohn’s disease is a rare manifestation of cutaneous Crohn’s disease. The literature on the diagnosis and treatment of this condition remains sparse, given its low prevalence. Herein, we present a case of a 47-year-old female with previously known inverse psoriasis and hidradenitis suppurativa. She was experiencing painful vulvar fissures, which ultimately resulted in a diagnosis of vulvar Crohn’s disease. The patient’s symptoms had resolved at the most recent follow-up visit with treatment, including adalimumab 40 mg, metronidazole 500 mg, and intralesional triamcinolone acetonide injections.

## Introduction

Crohn’s disease (CD) is a type of inflammatory bowel disease consisting of segmental granulomatous lesions that predominantly affect the ileum and colon, although any segment of the gastrointestinal tract may be involved.^1,2^ Extraintestinal features are present in ~40% of CD patients, with cutaneous involvement being the most common extraintestinal manifestation.^2^ Cutaneous involvement in CD typically occurs through bowel disease, contiguously extending to the skin.^1,2^ Cutaneous lesions with histopathological findings diagnostic of CD may also occur without any linkage to the gastrointestinal tract; this is known as metastatic CD or cutaneous CD (CCD).^1–3^ While gastrointestinal or perianal CD lesions may extend to involve the vulva, CD can similarly involve the vulva without a direct connection to intestinal CD manifestations.^1,3^ This specific extraintestinal manifestation of CCD is known as vulvar CD (VCD).^1,3,4^ VCD is exceptionally rare, with fewer than 200 cases documented in the literature.^3,4^ Optimal medical management of VCD remains an area of ongoing research.^4,5^

## Case report

**Figure 1.**
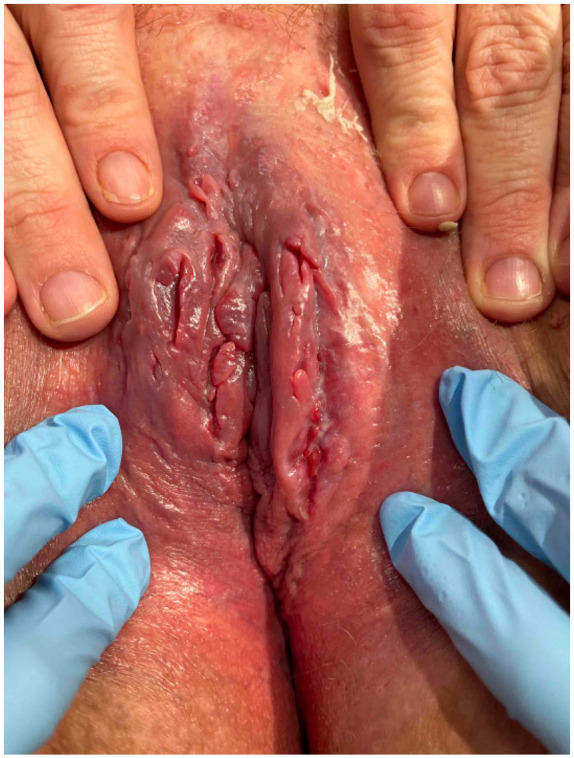
Initial presentation showing linear vulvar fissures, erythema, and swelling.

A 47-year-old premenopausal nulligravida female presented with a 2-year history of painful and pruritic linear vulvar fissures, erythema, and swelling (Figure 1). The patient was previously followed for biopsy-proven inverse psoriasis and hidradenitis suppurativa, both of which responded well to adalimumab. The patient also had recurrent anterior uveitis and was awaiting referral to rheumatology at the time of her initial presentation. There was no prior history of inflammatory bowel disease or related symptoms. Her current medications included adalimumab, chlorthalidone, perindopril, citalopram, zopiclone, lisdexamfetamine, and semaglutide. The patient was an office worker with a 20-pack-year smoking history, consumed two to three bottles of wine per week, and denied recreational drug use. She endorsed being sexually active despite dyspareunia and post-coital bleeding and had no history of sexually transmitted infections.

Fungal culture of the vulvar fissures was positive for Candida non-albicans yeast and group B *Streptococcus*, which we treated successfully with topical hydrocortisone/nystatin/zinc oxide paste, fluconazole 150 mg PO once, and cephalexin 500 mg PO QID for 7 days. As the fissures did not improve, we performed a punch biopsy of the vulva, which revealed dense lymphoplasmacytic infiltrate with multi-focal small non-caseating granulomas, which was highly suggestive of CCD. Though repeat fecal calprotectin was negative, we referred her to gastroenterology to rule out intestinal disease. We recommended continuing with adalimumab and prescribed metronidazole 500 mg PO twice daily. After 2 months of treatment, she was seen in follow-up with minimal improvement of the appearance of the fissures; however, she was experiencing less pruritus; the patient consented to intralesional triamcinolone acetonide 5 mg/mL injections (ILTAC) preceded by the application of lidocaine 5% gel. Six weeks later, both the appearance of the fissures as well as the associated pain and pruritus had improved, and ILTAC was repeated.

**Figure 2.**
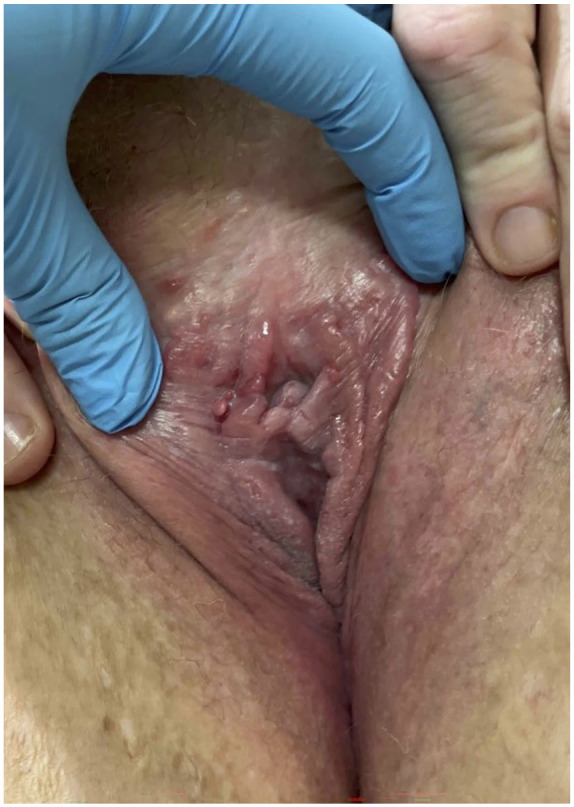
Resolution of vulvar fissures and symptoms at 22-month follow-up with adalimumab 40 mg, metronidazole 500 mg, and ILTAC injections.

Her fissures had completely resolved at the most recent follow-up with adalimumab 40 mg, metronidazole 500 mg, and ILTAC injections (Figure 2). In the interval, she had also been seen by gastroenterology for a colonoscopy, which revealed no evidence of intestinal CD.

## Discussion

Mucocutaneous manifestations of CD include dusky erythematous plaques with ulceration and undermining, linear knife-like ulcerations in the body folds or oral mucosa, draining sinuses, fistulas, and scarring.^2,3^ Reported genital findings include genital lymphedema leading to lymphangiectasias, edema, erythema, unilateral or bilateral labial swelling, pain or tenderness, hypertrophic lesions, chronic suppuration, skin tags, nodules, fissures, ulcers, and abscesses.^3,5–7^ Several cutaneous reactive disorders are associated with CD, including erythema nodosum, sweet syndrome, pyoderma gangrenosum, pathergy, pyostomatitis vegetans, necrotizing and granulomatous small vessel vasculitis, palisading neutrophilic granulomatous dermatitis, cutaneous polyarteritis nodosa, and epidermolysis bullosa acquisita.^2,6^ Hidradenitis suppurativa should also be kept on the differential, although not a cutaneous reactive disorder.^2^

The pathophysiology of CCD is mediated by TH1 and TH17, and elevated IL-17 and IL-23 levels.^2^ Mutations in *TRAF3IP2* are thought to be associated with CCD.^2^ Histopathology is classically characterized by non-caseating granulomas and chronic lymphocytic inflammation at cutaneous locations that are non-contiguous with the gastrointestinal tract.^8^

Our patient presented with VCD in the absence of any intestinal manifestations, including a negative colonoscopy and no prior history of inflammatory bowel disease. Up to 25% of patients present with vulvar lesions before a diagnosis of gastrointestinal CD is made.^9,10^ Further complicating the picture, patients in this group may experience nonspecific gastrointestinal symptoms that are often not investigated via endoscopy.^9^

Boxhoorn et al. proposed a diagnostic algorithm for VCD given the longstanding lack of consensus in the literature.^7,11^ For suspected VCD with supportive histological findings on vulvar biopsy but no gastrointestinal symptoms, the algorithm requires endoscopic features of CD to make a final diagnosis.^11^ We propose that the final diagnostic step of endoscopy is unnecessary to arrive at a diagnosis of VCD. This does not negate the importance of ruling out intestinal manifestations of CD nor the importance of long-term monitoring, given that many patients later develop intestinal CD^12^; however, active or prior intestinal manifestations of CD should not be necessary to initially diagnose VCD. VCD often takes years to diagnose and is frequently misdiagnosed; timely diagnosis may improve treatment outcomes and resource stewardship.^13^ Multidisciplinary care for patients with VCD remains essential, including but not limited to the involvement of dermatologists, dermatopathologists, gastroenterologists, and gynecologists.

Management of VCD primarily consists of topical corticosteroids or calcineurin inhibitors.^9^ Metronidazole, systemic corticosteroids, sulfasalazine, azathioprine, TNFα inhibitors, IL-12/IL-23 inhibitors, and surgical treatment options have also been used.^9,13,14^ Notably, our patient responded dramatically to ILTAC injections. Reporting on ILTAC as a treatment modality for VCD has been mixed and somewhat limited in the literature. A 2014 paper found that local corticosteroid injections were generally ineffective in managing VCD outside of a single case.^9^ A single patient in a 2015 retrospective review of 22 VCD cases was treated with ILTAC, of which a positive clinical response was observed.^15^ A 2020 case series of three VCD patients found varying responses to ILTAC injections, but the authors noted ILTAC to be an effective treatment modality overall.^8^ Notably, ILTAC injections remain absent from various proposed VCD treatment algorithms in the literature.^9,11,12^ Given the considerable improvement our patient experienced with ILTAC injections alongside several other encouraging recent reports in the literature, we believe that ILTAC should be considered a first-line therapeutic option for VCD patients. It should also be considered for patients who fail other treatment options and for persistent or recurrent cases before pursuing surgical management.

We present a case of VCD in the absence of intestinal manifestations with co-occurring hidradenitis suppurativa and psoriasis managed with adalimumab 40 mg, metronidazole 500 mg, and ILTAC. Despite the low prevalence, VCD should be included in the differential diagnosis for patients presenting with vulvar ulcers, regardless of the presence or absence of gastrointestinal symptoms. Furthermore, we recommend that physicians consider intralesional corticosteroid injections as a treatment for VCD, as this modality may rapidly improve symptoms and appearance.
